# Progress on implementing the WHO-GLASS recommendations on priority pathogen-antibiotic sensitivity testing in Africa: A *scoping review*

**DOI:** 10.12688/wellcomeopenres.23133.1

**Published:** 2024-11-22

**Authors:** Mackline Hope, Reuben Kiggundu, Dickson Tabajjwa, Conrad Tumwine, Fahad Lwigale, Herman Mwanja, J. P. Waswa, Jonathan Mayito, Daniel Bulwadda, Dathan M. Byonanebye, Francis Kakooza, Andrew Kambugu

**Affiliations:** 1 Infectious Diseases Institute, Makerere University, Kampala, Central Region, Uganda; 2 Management Sciences for Health Uganda, Kampala, Central Region, Uganda; 3 Medical Microbiology, College of Health Sciences, Makerere University, Kampala, Central Region, Uganda; 4 School of Public Health, Makerere University, Kampala, Central Region, Uganda

**Keywords:** Antimicrobial resistance; GLASS implementation; AMR Surveillance; Africa

## Abstract

**Introduction:**

The World Health Organization global antimicrobial resistance surveillance system (GLASS) was rolled out in 2015 to guide antimicrobial resistance (AMR) surveillance. However, its implementation in Africa has not been fully evaluated. We conducted a scoping review to establish the progress of implementing the WHO 2015 GLASS manual in Africa.

**Methods:**

We used MeSH terms to comprehensively search electronic databases (MEDLINE and Embase) for articles from Africa published in English between January 2016 and December 2023. The Arksey and O'Malley's methodological framework for scoping reviews was employed. Data were collected on compliance with WHO GLASS recommendations for AMR surveillance-priority samples, pathogens, and pathogen-antibiotic combinations and analysed using Microsoft Excel.

**Results:**

Overall, 13,185 articles were identified. 7,409 were duplicates, and 5,141 articles were excluded based on titles and abstracts. 609 full-text articles were reviewed, and 147 were selected for data extraction. Of the 147 selected articles, 78.9% had been published between 2020 and 2023; 57.8% were from Eastern Africa. 93.9% of articles were on cross-sectional studies. 96.6% included only one priority sample type; blood (n=56), urine (n=64), and stool (n=22). Of the 60 articles that focused on blood as a priority sample type, 71.7%, 68.3%, 68.3%, 36.7%, 30%, and 10% reported recovery of *Escherichia coli*, *Staphylococcus aureus, Klebsiella pneumoniae*, *Acinetobacter baumannii*, *Salmonella* species and *Streptococcus pneumoniae,* respectively. *Salmonella* and *Shigella* species were reported to have been recovered from 91.3% and 73.9% of the 23 articles that focused on stool. *E. coli* and *K. pneumoniae* recoveries were also reported from 94.2% and 68.1% of the 69 articles that focused on urine. No article in this review reported having tested all the recommended WHO GLASS pathogen-antibiotic combinations for specific pathogens.

**Conclusion:**

Progress has been made in implementing the GLASS recommendations in Africa, but adoption varies across countries limiting standardisation and comparability of data.

## Plain language

**Introduction:**

Antimicrobial resistance (AMR) is a growing problem globally, and it's particularly serious in Africa. To address this, the World Health Organization (WHO) created the Global Antimicrobial Resistance Surveillance System (GLASS). This study aimed to see how well African countries are following the GLASS guidelines.

**Methods:**

We looked at research papers published between 2016 and 2023 that collected data on AMR in Africa. We focused on studies that followed the GLASS guidelines and reported data on specific types of infections and bacteria.

**Results:**

We found that many African countries have made progress in implementing GLASS, but there are still gaps. Some countries are not reporting data on all the recommended infections and bacteria. Also, the quality of the data can be inconsistent.

**Discussion:**

The findings of this review highlight the progress made in implementing GLASS in Africa, but also identify significant gaps in the data collected and reported. The inconsistencies in pathogen-antibiotic combinations may be due to factors such as limited laboratory resources, lack of standardized methods, and variations in national guidelines.

Addressing these gaps is essential for improving the quality of AMR surveillance in Africa through investing in national AMR surveillance systems, developing standardized methods, and training laboratory staff. Collaboration between countries and international organisations is crucial for sharing data and best practices.

**Conclusion:**

While progress has been made, there is still work to be done to improve AMR surveillance in Africa. By addressing the gaps identified in this study, countries can better understand the extent of AMR and develop effective strategies to combat it.

## Introduction

Antimicrobial resistance (AMR) poses a significant threat to global public health and led to over 1.2 million deaths in 2019 ^1^. Low- and middle-income countries (LMICs) are disproportionately affected, probably due to the high burden of communicable diseases and lack of systems and resources for antimicrobial stewardship ^2^. While there is limited data on the geographical distribution, epidemiology, and impact of AMR, especially in the African region, conducting comprehensive population-based surveillance faces considerable challenges ^3– 6^. Further, assessing and monitoring AMR trends globally is limited due to the lack of quality data ^7^, especially on pathogens that pose significant public health risks ^8^.

AMR surveillance is fundamental ^3, 6^ and provides the basis for understanding the AMR burden ^9^. AMR surveillance data is critical for public health interventions, guides health policy decisions, and may unearth signals for emerging threats ^6^. In low- and middle-income countries (LMICs), the capability for AMR surveillance varies ^9^, with Sub-Saharan Africa (SSA) and Southeast Asia having the least developed coverage compared to high-income countries ^10– 12^. The low coverage in SSA is mainly due to weak infrastructure, limited resources, shortage of trained staff, and various socioeconomic factors ^13, 14^. Despite these challenges, several African countries have made strides towards the implementation of international guidelines, such as the World Health Organization (WHO) Global Antimicrobial Resistance Surveillance System (GLASS) ^15^, the Global Action Plan (GAP) and other WHO benchmarks ^16^.

Launched in 2015, the GLASS manual ^15^ aimed to guide the collection, analysis, and sharing of standardised AMR surveillance data to inform decision-making and drive action globally. The manual provides recommendations for the early implementation of AMR surveillance systems, including guidance on priority specimens, pathogens, and pathogen-antimicrobial combinations for monitoring resistance. The antimicrobial agents selected for AMR monitoring in each priority pathogen were chosen based on several criteria: they are either commonly recommended as first-line treatments, serve as surrogate markers for resistance to drugs frequently used in patient care, or represent pathogen-antimicrobial combinations of particular concern due to limited treatment options.

A detailed summary of the WHO-GLASS 2015 recommendations on priority specimens and antibiotic testing is provided in the extended data (Supplementary File 1) ^17^. The recommended priority specimens include blood, urine, faeces, urethral and cervical swabs (extended data, Supplementary File 2) ^17^, and these were selected because they are essential for isolating pathogens responsible for the most common bloodstream, urinary tract, gastrointestinal tract, and genital/reproductive tract infections ^15^. The purpose of using priority specimens and pathogens was to enable systematic capacity building for countries as they set up their national AMR surveillance programs ^18^. Despite these recommendations, countries could also report on additional specimens and pathogens if national capacity for surveillance of these organisms exists.

Although the GLASS manual was rolled out in 2015, the extent of its implementation, specifically for priority specimen and pathogen-antibiotic combinations, has not been fully explored, especially in Africa. We conducted a scoping review to establish the progress of implementation of the WHO GLASS manual 2015 in Arica. This review aimed to highlight gaps in knowledge and provide insights to guide current and future practices in AMR surveillance.

## Methods

This scoping review's ^19^ methodological framework was based on Arksey and O'Malley's scoping review process. This process which involves identifying research questions for the review; a comprehensive search to identify relevant studies; selection of eligible studies based on clear inclusion and exclusion criteria; extraction and organisation of data from selected studies; and summarising and reporting of the results ^20^. It was reported following the Preferred Reporting Items for Systematic Reviews and Meta-Analyses Extension of Scoping Review (PRISMA-ScR) ^21^. No institutional review board (IRB) protocol clearance was required since it was based on already published articles, and no patients or the public were directly involved in the study.

### Eligibility criteria

The review focused on peer-reviewed journal articles from Africa that focused on priority specimens, specifically blood, urine, and stool, as well as the appropriate priority pathogens, pathogen-antibiotic combinations and reporting in following the "susceptible (S)", "intermediate (I)", and "resistant (R)" categories as recommended by GLASS. The papers were included if they: had been published between January 2016 and December 2023; involved data collection between 2016 and 2023; were written in English; involved human samples; and described antimicrobial sensitivity testing as per WHO GLASS priority specimen and priority pathogens recommendations. We excluded non-peer-reviewed articles (editorials, commentaries), review papers and any other publication without primary data, articles written in other languages and those with insufficient data regarding the study question.

### Search strategy

We searched the following electronic databases for academic articles from 2016 to December 2023: MEDLINE and EMBASE via Ovid (searches exclusive of pre-print studies) completed on 30th March 2024. These databases are considered reliable and comprehensive sources of academic literature across disciplines ^22^. The Keywords used for the search included: “Antimicrobial Resistance”, “Anti-microbial Susceptibility”, “AST”, “AMR Surveillance”, “Diagnostic”, “Africa”, and specific names of all African countries.

### Selection of sources of evidence

The search strategy (extended data, Supplementary File 3 ^17^) was developed by an experienced knowledge management specialist (RK) and further refined by HM and DT through team discussion. The final search results were exported into EndNote 21.3 ^23^ to manage citations. Two reviewers (HM and DT) screened the titles, abstracts, and full texts retrieved through a web-based collaboration software, Covidence, to identify potential articles for eligibility and inclusion in the scoping review. Conflicts were resolved by RK.

### Data extraction

We developed a data extraction form in Microsoft Excel (Microsoft Corporation, USA) for data management and processing ^24^. The customised data extraction tool collected relevant information on: i) key study characteristics (e.g. author, publication year, study title, study design, study setting, country, patient population characteristics); ii) detailed information on the study context and population; iii) and the Antimicrobial Susceptibility Testing (AST) of bacterial isolates according to the WHO GLASS recommendations for appropriate pathogen antibiotic combinations and reporting as Resistant (R), Intermediate (I) and Sensitive (S). At the same time, reviewers documented reasons for the exclusion and identified those articles considered relevant and eligible.

A calibration exercise was undertaken to ensure systematic and reproducible study selection and data extraction processes. First, the review lead (RK) used a seminal article to ascertain if the extraction tool was appropriate for its intended use. Once confidence in the tool had been established, authors (paired MH – HM, CT – DT and HM - FL) piloted and reviewed a sample of twenty papers, discussing any questions and further refining the extraction tool.

Articles confirmed for inclusion in the scoping review were then moved to the final data extraction, charting, and synthesis stages. For data extraction and review, author pairs (MH – HM, CT – DT and HM - FL) independently extracted data in duplicate from each eligible article. All disagreements among the reviewers were resolved through discussion. The supervising reviewer (RK) resolved any conflicts and provided oversight for the whole process.

All data reported in the included articles were reviewed to check if the AST was performed in accordance with the WHO GLASS recommended priority pathogen-antibiotic combinations and reported as per the "susceptible", "intermediate", and "resistant" recommendations of the Clinical & Laboratory Standards Institute (CLSI) or the European Committee on Antimicrobial Susceptibility Testing (EUCAST). Consistent with the JBI scoping review methodology, MH, HM, CT, DT, and FL used the Critical Appraisal Skills Programme (CASP) checklist ^25^ to critically appraise the research articles for quality, (extended data, Supplementary File 4 ^17^).

### Data synthesis and analysis

Our descriptive analysis conducted by FL and MH explored key characteristics of the articles reviewed, such as publication year, study country described per African Region, study setting, study types, study population, and WHO priority specimen. Furthermore, data on the progress of the WHO GLASS implementation was stratified by WHO Priority specimen/s and reported per priority pathogen–antibiotic combination. We used tabular and thematic strategies to summarise the extracted data.

## Results

**Figure 1.**
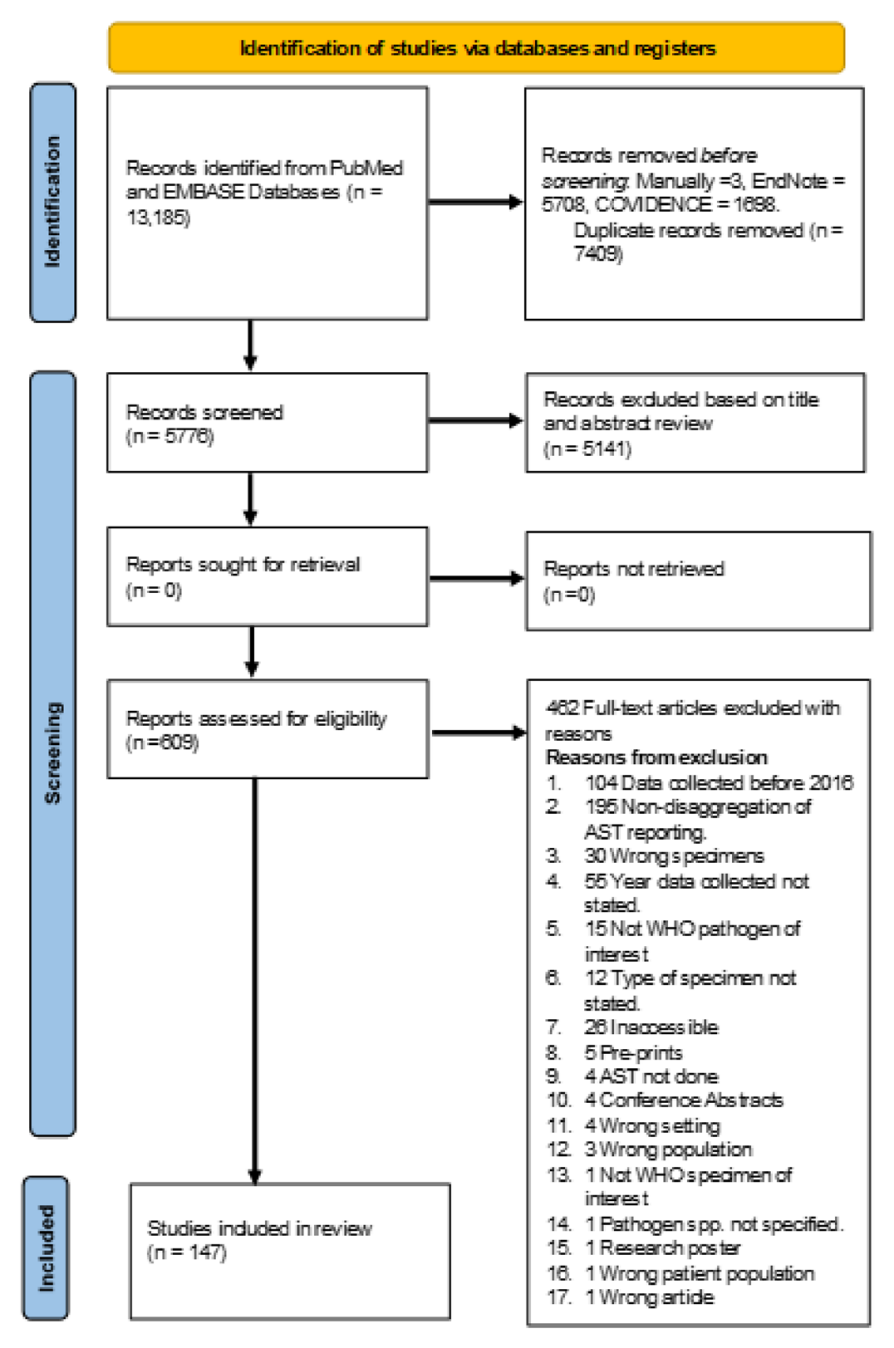
PRISMA-ScR flow diagram for the process of study selection.

After conducting a comprehensive literature search, 13,185 articles were identified; 7,409 duplicates were removed. After screening the titles and abstracts, 5141 articles were excluded. Subsequently, 609 articles underwent a full-text review, from which 147 were selected for further data extraction ( Figure 1).

Of the 147 included articles, 116 (78.9%) were published between 2020 and 2023 and 85 (57.8%) were from Eastern Africa ( Table 1). Other articles were from the Western, 35 (23.8%); Northern, 15 (10.2%); Southern, 7 (4.8%); and Central, 5 (3.4%) African subregions. Notably, Ethiopia had the highest number of articles 49 (31.9%). The majority of the articles, 138 (93.9%), were on cross-sectional studies. 142 (96.6%) articles included only one priority sample type—: blood, 56 (38.1%); urine, 64 (43.5%); and stool, 22 (15.0%). 5 (3.4%) articles included two priority sample types, mainly urine and blood, 4 (2.7%).

**Table 1.** Characteristics of included articles.

Characteristic	Number, (n=147)	Percentage (%)
**Years of publication for** ** the included papers**		
2016–2019	39	21.1
2020–2023	116	78.9
African Regions * where ** the included articles** ** were conducted**		
Central Africa	5	3.4
Eastern Africa	85	57.8
Northern Africa	15	10.2
Southern Africa	7	4.8
Western Africa	35	23.8
**Study settings of the** ** included papers**		
Home/Community	5	3.5
Clinic/Hospital	142	96.6
**Study types of the** ** included papers**		
Cross-sectional	138	93.9
Cohort	7	4.8
Others	2	1.4
**Study population**		
General population	13	8.8
Clinical **	134	91.2
**GLASS priority** ** specimen considered in** ** the study**		
**Only 1 specimen type**		
Blood	56	38.1
Urine	64	43.5
Stool	22	15.0
**2 specimen types**		
Blood and urine	4	2.7
Urine and stool	1	0.7
**All three specimen** ** types**	0	0.0

*GLASS: Global Antimicrobial resistance Surveillance System* *African Regions as per the African Union geographical regions **Clinical study population refers to studies conducted among patients.

Among the 60 articles that reported blood as one of the priority specimens, 14 (23.3%), 11 (18.3%), 12 (20%), 17 (28.3%), 5 (8.3%), and 1 (1.7 %) recovery of one, two, three, four, five and six priority pathogens, respectively. Up to 65.2% of the 23 articles that reported stool and 62.3% of the 69 articles that reported urine as priority specimens had recovery of two priority pathogens.

Of the 60 articles that focused on only blood as a priority sample type 43 (71.7%), 41 (68.3%), 41 (68.3%), 22 (36.7%), 18 (30%), and 6 (10%) reported the recovery of *Escherichia coli*, *Staphylococcus aureus, Klebsiella pneumoniae*, *Acinetobacter baumannii*, *Salmonella* species and *Streptococcus pneumoniae* respectively. *Salmonella and Shigella* species were reported to have been recovered from 91.3% and 73.9% of the 23 articles that focused on only stool, respectively. *Escherichia coli* and *Klebsiella pneumoniae* recoveries were also reported from 94.2% and 68.1% of the 69 articles that focused on only urine, respectively, Table 2. The disaggregation of the priority pathogens recovered across the papers retrieved in this review is shown in Table 3.

**Table 2.** Priority pathogens recovered in included articles.

Priority pathogen recovery across the papers	Number of articles *	Percentage (%)
**Blood**	n=60	
*Acinetobacter baumannii*	22	36.7
*Escherichia coli*	43	71.7
*Klebsiella pneumoniae*	41	68.3
*Staphylococcus aureus*	41	68.3
*Streptococcus pneumoniae*	6	10.0
*Salmonella* spp.	18	30.0
**Stool**	n = 23	
*Salmonella* spp.	21	75.0
*Shigella* spp	17	60.2
**Urine**	n=69	
*Escherichia coli*	65	94.2
*Klebsiella pneumoniae*	47	68.1

*Articles included in this analysis had one or two (2) priority specimens.

**Table 3.** Disaggregation of the WHO GLASS priority pathogens reported to have been recovered across the included articles.

Priority pathogen recovery per priority specimen	Number of Studies	References
**Blood**		
**Only one priority pathogen**		
only *A. baumannii*	3	26– 28
only *E. coli*	1	29
only *K. pneumoniae*	5	30– 34
only *S. aureus*	2	35, 36
only *S. pneumoniae*	0	
only *Salmonella* spp.	4	37– 40
two priority pathogens	11	41– 51
three priority pathogens	12	52– 63
Four priority pathogens	17	64– 80
Five priority pathogens	5	81– 85
Six priority pathogens	1	86
**Stool**		
**Only one priority pathogen**		
*Salmonella* spp.	6	87– 92
*Shigella* spp	2	93, 94
Both *Salmonella* spp and *Shigella* spp	15	95– 109
**Urine**		
**Only one priority pathogen**		
*E. coli*	22	110– 131
*K. pneumoniae*	3	31, 32, 132
*E. coli* & *K pneumoniae*	44	30, 59, 73, 87, 133– 172

All evaluated articles appropriately reported final AST results in terms of S, I, and R as per the WHO GLASS recommendations.

### Conformity of articles to the WHO GLASS pathogen – antibiotic combination per priority specimen

Generally, 97.7% (43) of the 44 pathogen-antibiotic combinations reported in the retrieved articles didn’t conform to the WHO GLASS recommendations, irrespective of the priority specimen. Table 4 shows compliance of the 44 WHO GLASS priority pathogen-antibiotic combinations in the included articles with WHO GLASS recommendations.

**Table 4.** Compliance of the 44 WHO GLASS priority pathogen-antibiotic combinations in the included articles with WHO GLASS recommendations.

	Priority specimen	Blood						Stool		Urine	
	Priority pathogen	*A. baumanii* (n=22)	*E. coli* (n=43)	*K. pneumoniae* (n=41)	*S. aureus* (n=41)	*S. pneumonia* (n=6)	*Salmonella* **spp.**(n=18)	*Salmonella* **spp.**(n=21)	*Shigella* **spp**n=17)	*E. coli* (n=65)	*K. pneumoniae* (n=47)
**Antibiotics**	**Tetracyclines** (Tigecycline or minocycline)	5	N/A	N/A	N/A	N/A	N/A	N/A	N/A	N/A	N/A
	**Aminoglycosides** (Gentamicin and amikacin)	10	N/A	N/A	N/A	N/A	N/A	N/A	N/A	N/A	N/A
	**Carbapenems** (Imipenem, ertapenem, meropenem or doripenem)	17 ^c^	32	34	N/A	N/A	10	5	N/A	44	33
	**Polymixins** (Colistin)	5	3	5	N/A	N/A	N/A	N/A	N/A	5	2
	**Sulfonamides and** ** trimethoprim** (Co- trimoxazole)	N/A	29	29	N/A	3	N/A	N/A	N/A	50	35
	**Floroquinolones** Ciprofloxacin or levofloxacin.	N/A	36	36	N/A	N/A	16	21	16	57	43
	**Third-generation** ** cephalosporins**	N/A	28	29	N/A	3	7	6	5	43	30
	(Ceftriaxone or cefotaxime and ceftazidime)										
	**Fourth-generation** ** cephalosporins** (Cefepime)	N/A	13	13	N/A	N/A	N/A	N/A	N/A	16	12
	**Penicillins** (Ampicillin)	N/A	32	N/A	N/A	N/A	N/A	N/A	N/A	44	N/A
	**Penicillinase-** **stable beta-** **lactams** (Cefoxitin ^a^/Oxacillin ^b^)	N/A	N/A	N/A	20	N/A	N/A	N/A	N/A	N/A	N/A
	**Penicillins** (Oxacillin ^b^)	N/A	N/A	N/A	N/A	0	N/A	N/A	N/A	N/A	N/A
	**Penicillins** (Penicillin G)	N/A	N/A	N/A	N/A	5	N/A	N/A	N/A	N/A	N/A
	**Macrolides** (Azithromycin)	N/A	N/A	N/A	N/A	N/A	N/A	N/A	5	N/A	N/A

Note: ^a^ Cefoxitin is a surrogate for testing susceptibility to oxacillin (methicillin, nafcillin). ^b^ Oxacillin is a surrogate for testing reduced susceptibility or resistance to penicillin. ^c^ Ertapenem not among carbapenems set for organism in accordance to GLASS. N/A: Not applicable.

### Blood

Among the 22 articles that reported blood culture results with *Acinetobacter baumannii*, 5 (23%) adhered to the GLASS pathogen antibiotic guidelines for testing tetracyclines and polymixins, 10 (45%) for aminoglycosides, and 17 (77%) for carbapenems.

Forty-nine percent (20/49) of the articles that reported the recovery of *Staphylococcus aureus* from blood followed the GLASS recommendations for using penicillinase-stable beta-lactams to detect Methicillin-Resistant *Staphylococcus aureus* (MRSA).

Among the 43 articles that documented the culture of blood samples with recovery of *Escherichia coli*, the conformance to GLASS recommendation was recorded in 36 (84%) for fluoroquinolones, 32 (74%) for penicillins and carbapenems, 29 (67%) for sulfonamides/trimethoprim, 28 (65%) for third-generation cephalosporins, 13 (30%) for fourth-generation cephalosporins, and 3 (7%) for polymyxins.

Of the 41 articles that documented culture of blood samples with the recovery of *K. pneumoniae*, 36 (88%) followed the GLASS prioritisation of fluoroquinolones for AST, 32 (83%) for carbapenems, 29 (71%) for sulfonamides/trimethoprim and third-generation cephalosporins, 13 (32%) for fourth-generation cephalosporins, and 5 (12%) for polymyxins.

From the 18 articles that reported the recovery of *Salmonella* spp from blood, 16 (89%) showed conformance to the GLASS pathogen-antibiotic recommendation for fluoroquinolones, 10 (56%) for carbapenems, and 7 (39%) for third-generation cephalosporins.

Additionally, out of the 6 articles that reported recovery of *Streptococcus pneumoniae* from blood, the conformance to GLASS prioritisation of antibiotics for AST was recorded in 3 (50%) for sulfonamides/trimethoprim and third-generation cephalosporins and 5 (83%) for penicillins (Penicillin G).

### Stool

The 21 articles that reported the recovery of *Salmonella* spp from stool samples were 100% conformant to the GLASS prioritisation of fluoroquinolones. The conformance to the GLASS prioritisation for carbapenems and third-generation cephalosporins was registered among 5 (24%) and 6 (29%) articles, respectively.

Of the 17 articles that reported *Shigella* spp recovery from stool, 16 (94%) reported conformance to the GLASS prioritisation for fluoroquinolones whereas 5 (29%) reported conformance for macrolides and third-generation cephalosporins.

### Urine

Out of the 65 articles that documented culture of urine samples with the recovery of *Escherichia coli*, 57 (88%) showed conformance GLASS prioritisation of antibiotics for AST for fluoroquinolones, 50 (77%) for sulfonamides/trimethoprim, 44 (68%) for penicillins and carbapenems, 43 (66%) for third-generation cephalosporins, 16 (25%) for fourth-generation cephalosporins, and 5 (8%) for polymyxins.

Among the 47 articles that documented *Klebsiella pneumoniae* recovery from urine samples, 43 (91%) followed GLASS prioritisation for fluoroquinolones, 35 (74%) for sulfonamides/trimethoprim, 33 (70%) for carbapenems, 30 (64%) for third-generation cephalosporins, 12 (26%) for fourth-generation cephalosporins, and 2 (4%) for polymyxins.

## Discussion

In this review, of the adoption of the WHO GLASS recommendations in Africa, we found 147 articles. Our review highlights significant inconsistencies in the pathogen-antibiotic combinations across the different articles. In majority of the articles, urine is a predominant priority sample, followed by blood and stool. To the best of our knowledge, this is the first comprehensive review of the progress of African states in complying with the GLASS recommendations. This review highlights that urine is the predominant priority sample collected, followed by blood and stool. This might be attributable to the fact that urinary tract infections (UTIs) are the most common infectious diseases diagnosed worldwide, particularly in developing countries ^173^. The distinct recovery of *E. coli* from urine in the articles in this review is similar to that seen in prior reviews on the African continent ^174, 175^.

Other evidence from Africa shows that bloodstream infections are quite prevalent (up to 14.6%) and associated with a high disease burden, including death ^176– 179^. These infections have mainly been attributed to *Salmonella* spp, *Streptococcus pneumoniae*, *Staphylococcus aureus*, and *Escherichia coli* pathogens ^180^. Similar organisms were found to be responsible for most bloodstream infections as found in this review. Several reviews and studies on the African continent have indicated a low prevalence of *Salmonella* and *Shigella* spp infections, consistent with the findings in this review ^181– 183^.

This review showed significant inconsistencies in the pathogen-antibiotic combinations across the different articles retrieved. These inconsistencies were not different from those found in other regions, such as the Middle East, where substantial heterogeneity in pathogen-antibiotic combinations has been reported ^184^. The lack of conformity for most combinations of pathogens and antibiotics may be attributed to the fact that most AMR data in Africa are collected through inadequate laboratory-based surveillance systems that form a part of routine patient care rather than comprehensive national surveillance systems ^9, 185, 186^. The non-conformance could also be attributable to non-compliance to the standard CLSI and EUCAST guidelines for AST across Africa ^15, 187^. The limited observation of the quality assurance measures in microbiology laboratories in many low-resource settings ^188, 189^ further exacerbates the non-conformance ^190^. The resource limitations in many African countries ^191, 192^ and weak supply chains for consumables for microbiological laboratory procedures ^193^, such as AST, equally hinder the implementation of the GLASS pathogen-antibiotic prioritisation. The limited AMR surveillance methods and reporting protocols impede the usefulness, validity and trustworthiness of the data generated from the surveillance systems on the continent ^194^. Additionally, the absence of national action plans (NAP) for AMR and implementation roadmaps for GLASS in some countries further hinders adherence to the GLASS recommendations ^195^.

Non-conformance for pathogen-antibiotic combination tested against polymyxin (using conventional methods) is likely attributed to the current CLSI ^196^ or EUCAST guidelines that recommend minimal inhibitory concentration (MIC) determination methods for AST, which are not routinely accessible by the majority of the AMR surveillance sites in LMICs. Additionally, automated platforms such as VITEK 2 and BD PHENIX M50 for AST (MIC) require highly skilled personnel and are associated with high purchase, reagent and maintenance costs ^197^, which are not widely available in the African setting ^192^.

To tackle these challenges, various regional and national associations and networks are supporting laboratories in Africa to strengthen national laboratory AMR systems ^198, 199^. One such organisation is the African Society for Laboratory Medicine (ASLM), which advocates for the adoption of the Stepwise Laboratory Quality Improvement Process Towards Accreditation (SLIPTA) framework ^185, 199^. SLIPTA aims to enhance the evaluation and verification of the establishment, implementation and improvement of quality management systems in health laboratories ^200, 201^.

Despite this study's positive outcomes and strengths, it still had limitations. This scoping review was conducted with reference to the first WHO GLASS Manual, 2015, whose scope was still low. Further evaluation could be necessary considering the updated WHO GLASS Manual 2.0. Secondly, there was no inclusion of grey literature sources such as country and WHO reports or articles written in other languages besides English, which could have provided more insights Urethral and cervical swabs targeting *N. gonorrhoae* were not considered since a recent study comprehensively evaluated them ^202^.

## Conclusion and recommendations

This scooping review shows that despite a few shortfalls, there has been significant progress in implementing the WHO GLASS manual recommendations across Africa. This deviation underscores gaps in the implementation of GLASS guidelines and highlights the urgent need for improved strategies to align current practices with global antimicrobial resistance surveillance objectives. These findings also emphasise the necessity for streamlining efforts to address the identified deficiencies to enhance conformity and ensure more robust and effective AMR monitoring and reporting systems worldwide. Furthermore, capacity building is recommended, including training laboratory personnel about AST guidelines and increasing access to MIC determination methods.

## Ethics and consent

Ethical approval and consent were not required.

## Abbreviations

**Table:** 

AMR	Antimicrobial Resistance
AST	Antimicrobial Susceptibility Testing
CLSI	Clinical & Laboratory Standards Institute
EUCAST	European Committee on Antimicrobial Susceptibility Testing
GAP	Global Action Plan
GLASS	Global Antimicrobial Resistance Surveillance System
NAP	National Action Plan
PRISMA-ScR	Preferred Reporting Items for Systematic Reviews and Meta-Analyses Extension of Scoping Review
WHO	World Health Organisation

## Data Availability

No data are associated with this article. The extended data utilised for this review is available in the Open Science Framework (OSF) titled “Progress on implementing the WHO-GLASS recommendations on priority pathogen-antibiotic sensitivity testing in Africa: A Scoping Review”, https://doi.org/10.17605/OSF.IO/4KW9Z ^17^. The data is available for non-commercial use under a Creative Commons (CC0) 1.0 Universal.
