# Supplementary material for: Progress on implementing the WHO-GLASS recommendations on priority pathogen-antibiotic sensitivity testing in Africa: A scoping review
Source: Wellcome Open Res. 2024 Nov 22;9:692. [Version 1] doi: 10.12688/wellcomeopenres.23133.1 (PMC11809157; doi:10.12688/wellcomeopenres.23133.1)
Supplement: Peer review history

# Reviewer report: Reviewer response for version 1

**DOI:** 10.21956/wellcomeopenres.25473.r113214

**Published:** 2025-01-21

**Authors:** Sara Tomczyk

**Affiliations:** 1 Robert Koch Institute, Berlin, Germany

This is a useful review contributing to the important ongoing challenge, namely the lack of quality-assured AMR results in the African region. However, it is lacking some important reported results from the included articles as well as discussion of relevant considerations/context as discussed below.

**Major:**

**INTRODUCTION:**

- It is important to note that there is a revised GLASS manual that was published in August 2023 overlapping with your search time period until December 2023. At a minimum, it should be mentioned in the introduction with a couple sentences on the key differences e.g. in the priority specimens, pathogens and antibiotic testing as well as in the discussion in the paper on how your results compared also to the updated current manual recommendations.
- The extent of GLASS implementation, including in Africa, has been explored and presented by WHO through reported implementation indicators, e.g. on its dashboard: Global AMR data. I think it is still worthwhile to have explore its implementation through other sources identified in your review, but you should compare to the WHO GLASS implementation indicator/results findings for African countries. Perhaps a short mention in the introduction, a comparative table in the results and then more text on the key comparative findings in the discussion.

**METHODS:**

- In your search strategy, one could have considered the alternatives terms for AMR and testing, e.g. "antibiotic resistance", "sensitivity testing", otherwise some manuscripts may have been missed.
- In the data extraction, did you also collect information on pathogen identification/manual culture vs other automated methods, use of quality control (or QM/QA), and use of breakpoint guidelines for AST (e.g. EUCAST, CLSI). These are common reasons for different results and poor quality of results. It would have also been interesting to know if all were isolate-based/lab-based results reported or if any were patient-based.

**RESULTS:**

- I suggest clearer distinguishing which papers were reported as "surveillance results" and/or noted the use of or reporting to GLASS.
- I think some data extraction and summary of results concerning the type of methods used for pathogen identification and AST reported in the manuscripts is needed in order to better interpret the pathogen-antibiotic findings.
- I suggest listing the results of reported Gram-negative pathogens first and then followed by Gram positive pathogens.
- In Table 3, please include %.
- In Table 4, I wonder if "compliance" is the best term for the title. Perhaps simply "Reported testing results of the 44 WHO GLASS...." to be clearer. Please also add % in the cells for clearer reading of the table.
- In table 4, it would also be interesting to know of the included articles, which ones should AST results where there are actually no EUCAST/CLSI breakpoints (so really likely no recommended to be tested at all and/or unclear how they determined the SIR results if there are no breakpoints). Actually, this proportion of noncompliance would be an important finding.
- Did any of the paper report contamination rates? This would also be quite interesting and important to report.
- As mentioned above, I do think a comparison of these results to the summary published by WHO on its GLASS implementation dashboard for African countries would be quite valuable and put your findings more into context.

**DISCUSSION:**

- First paragraph: You state that the fact that urine was the sample findings most often found could be attributed to the fact that UTIs are commonly diagnosed globally is just one contributing factor. Another common factor is that urine culture is much easier to collect, and test compared to blood culture where pathogen detection rates are quite low.
- Second paragraph: I think you really need to consider the challenges of implementing quality-assured blood culture diagnostics which affects results. Please review this article which reviews important considerations and feasibility: Ombelet S, *et al.* 2019 ^1^

- As mentioned above, please consider noting in the discussion the publication of the 2023 GLASS manual, the reported GLASS implementation dashboard findings and any important comparisons here to your findings.

**Minor:**

**OVERALL:**

- In general, the manuscript would benefit from another review of English language editing as there are some sentences that are not written grammatically correct, or the flow of the sentence could be slightly improved.

**INTRODUCTION:**

- First paragraph: I find it important to note that LMICs are disproportionately affected also due to lack of systems and resources for diagnostic stewardship, e.g. "lack of systems and resources for diagnostic and antimicrobial stewardship." ALSO, the word "data" is always plural! E.g. "While there are limited data on the..." Please check the manuscript throughout for this.
- Second paragraph: I think it's important to insert the word "often" into the sentence "with Sub-Saharan Africa (SS) and Southeast Asia often having the least developed coverage compared to high-income countries" since it is not always the case, e.g. other low-resource settings in the West Pacific or the Americas also have similar challenges.
- Fifth paragraph: In, "WHO GLASS manual 2015 in Arica", Africa is misspelled.

Are the rationale for, and objectives of, the Systematic Review clearly stated?

Yes

Is the statistical analysis and its interpretation appropriate?

Partly

If this is a Living Systematic Review, is the ‘living’ method appropriate and is the search schedule clearly defined and justified? (‘Living Systematic Review’ or a variation of this term should be included in the title.)

Not applicable

Are sufficient details of the methods and analysis provided to allow replication by others?

Partly

Are the conclusions drawn adequately supported by the results presented in the review?

Partly

Reviewer Expertise:

Bacteriology, antimicrobial resistance, surveillance, epidemiology, infection prevention and control

I confirm that I have read this submission and believe that I have an appropriate level of expertise to confirm that it is of an acceptable scientific standard, however I have significant reservations, as outlined above.

## References

[1] : Best Practices of Blood Cultures in Low- and Middle-Income Countries. *Front Med (Lausanne)* .2019;6: 10.3389/fmed.2019.00131 131 10.3389/fmed.2019.00131 31275940 PMC6591475

# Reviewer report: Reviewer response for version 1

**DOI:** 10.21956/wellcomeopenres.25473.r113215

**Published:** 2024-12-26

**Authors:** Jens Thomsen

**Affiliations:** 1 Public Health and Epidemiology, Khalifa University, Abu Dhabi, Abu Dhabi, United Arab Emirates

This is a well written manuscript, adding value to the scientific body of literature on the implementation status of GLASS AMR surveillance manual and related  protocols in the African continent.  Acceptance of the article is strongly recommended.

**Some minor comments:**

**INTRODUCTION:**

 First sentence: "Antimicrobial resistance (AMR) poses a significant threat to global public health and led to over 1.2 million deaths in 2019." Here the 2019 GBD study published in 2022 is cited. This citation can be replaced by the updated GBD study, published in Sep 2024 (Lancet 2024 Sep 28;404(10459):1199-1226.  Doi: 10.1016/S0140-6736(24)01867-1. Epub 2024 Sep 16.) Accordingly, "1.2 million deaths in 2019" should be replaced by "1.14 millions deaths attributable to bacterial AMR, and 4.71 million deaths associated with bacterial AMR in 2021."

 4th paragraph: "Despite these recommendations, countries could also report on additional specimens and pathogens if **considered a national priority and **capacity for surveillance of these organisms exists."

**METHODS:**

 1st paragraph: "This process involves ..." (delete the word "which")

 Search strategy:

 As the manuscript focuses much on GLASS, why was the term "GLASS" not included in the search terms?

 Furthermore, GLASS is aiming at guiding AMR surveillance at the national level. Why was the term "national" not included in the search terms?

 It is not clear if the articles searched for are primarily on local (e.g. hospital), or national AMR surveillance efforts, or both? Further clarification in this regard might be helpful. 

 RESULTS:

 3rd paragraph: "... and 1 (1.7%) **reported** recovery of ..." (there seems to be a word missing)

**DISCUSSION:**

 4th paragraph: Change "BD PHENIX" to "BD PHOENIX"

 6th (last) paragraph: "... provided more insights." (add a dot to end the sentence)

**REFERENCES:**

 To replace the 1st reference (Murray CJ et al) by the updated GBD study published in Sep 2024 in Lancet  (see above)

 --- end of report ---

Are the rationale for, and objectives of, the Systematic Review clearly stated?

Yes

Is the statistical analysis and its interpretation appropriate?

Yes

If this is a Living Systematic Review, is the ‘living’ method appropriate and is the search schedule clearly defined and justified? (‘Living Systematic Review’ or a variation of this term should be included in the title.)

Yes

Are sufficient details of the methods and analysis provided to allow replication by others?

Yes

Are the conclusions drawn adequately supported by the results presented in the review?

Yes

Reviewer Expertise:

National AMR Surveillance programs

I confirm that I have read this submission and believe that I have an appropriate level of expertise to confirm that it is of an acceptable scientific standard.

## References

[1] Global burden of bacterial antimicrobial resistance 1990–2021: a systematic analysis with forecasts to 2050 Naghavi, Mohsen et al. The Lancet, Volume 404, Issue 10459, 1199 - 1226.10.1016/S0140-6736(24)01867-1PMC1171815739299261

# Reviewer report: Reviewer response for version 1

**DOI:** 10.21956/wellcomeopenres.25473.r113210

**Published:** 2024-12-13

**Authors:** Samuel Kariuki

**Affiliations:** 1 Kenya Medical Research Institute, Nairobi, Kenya

This is a very well researched and clearly written systematic review that sought to review progress made in the implementation of National Action Plans mitigating against AMR for Africa countries, challenges and opportunities for improvement in reporting data to WHO-GLASS platform.

 A number of important challenges were observed, including inconsistencies in pathogen antibiotic combinations which may be due to factors such as limited laboratory resources, lack of standardized methods, and variations in national guidelines.

  While progress has been made, there is still work to be done to improve AMR surveillance in Africa and by addressing the gaps identified in this study, countries can better understand the extent of AMR and develop effective strategies to combat AMR.

**A few Minor Corrections:**

**ABSTRACT**

- **Page 1, Line:** We conducted a scoping review to establish the progress of implementing the WHO 2015 GLASS manual in Africa.
- **Comment:** Manual or Guidelines? Clarify, as these two terms have different interpretations.
- **Page 3, Line:** While progress has been made, there is still work to be done to improve AMR surveillance in Africa. By addressing the gaps identified in this study, countries can better understand the extent of AMR and develop effective strategies to combat it.
- **Comment:** Use burden of AMR instead

**INTRODUCTION**

- **Page 10, Line:** EUCAST guidelines for AST across Africa. The limited observation of the quality assurance measures in microbiology laboratories in many low-resource settings, further exacerbates the non-conformance. The resource limitations in many African countries, and weak supply chains for consumables for microbiological laboratory procedures, such as AST, equally hinder the implementation of the GLASS pathogen antibiotic prioritisation. The limited AMR surveillance methods and reporting protocols impede the usefulness, validity and trustworthiness of the data generated from the surveillance systems on the continent. Additionally, the absence of national action plans (NAP) for AMR and implementation roadmaps for GLASS in some countries further hinders adherence to the GLASS recommendations.
- **Comment:** Several limitations to effective surveillance and data on AMR highlighted here, these are important points for intervention to work and require dissemination strategy to appropriate authorities.

Are the rationale for, and objectives of, the Systematic Review clearly stated?

Yes

Is the statistical analysis and its interpretation appropriate?

Yes

If this is a Living Systematic Review, is the ‘living’ method appropriate and is the search schedule clearly defined and justified? (‘Living Systematic Review’ or a variation of this term should be included in the title.)

Yes

Are sufficient details of the methods and analysis provided to allow replication by others?

Yes

Are the conclusions drawn adequately supported by the results presented in the review?

Yes

Reviewer Expertise:

Epidemiology and Genomics of Enteric pathogens and AMR

I confirm that I have read this submission and believe that I have an appropriate level of expertise to confirm that it is of an acceptable scientific standard.

# Response

**Published:** 2025-01-09

**Authors:** Mackline Hope

**Affiliations:** Research Department, Makerere University Infectious Diseases Institute, Kampala, Central Region, Uganda

**Responses to the comments** Thank you for the constructive comments. Below are the responses.

**Comment 1:** Manual or Guidelines? Clarify, as these two terms have different interpretations. **Response:** The GLASS manual was utilised.  

**Comment 2:** Use burden of AMR instead

**Response:** Burden of AMR is okay to make the statement read as “By addressing the gaps identified in this study, countries can better understand the burden of AMR and develop effective strategies to combat it.”  

**Comment 3:** Several limitations to effective surveillance and data on AMR highlighted here, these are important points for intervention to work and require dissemination strategy to appropriate authorities.

**Response:** This has been noted. We plan on developing appropriate recommendations and interventions following this work.
